# Assessment of Myocardial Viability in Ischemic Cardiomyopathy With Reduced Left Ventricular Function Undergoing Coronary Artery Bypass Grafting

**DOI:** 10.1002/clc.24307

**Published:** 2024-07-02

**Authors:** Arian Arjomandi Rad, Eleni Tserioti, Dimitrios E. Magouliotis, Robert Vardanyan, Ilias V. Samiotis, John Skoularigis, Ben Ariff, Andrew Xanthopoulos, Filippos Triposkiadis, Roberto Casula, Thanos Athanasiou

**Affiliations:** ^1^ Division of Medical Sciences University of Oxford Oxford UK; ^2^ Department of Surgery and Cancer Imperial College London London UK; ^3^ Department of Cardiothoracic Surgery University Hospital of Larissa Larissa Greece; ^4^ Department of Cardiology University Hospital of Larissa Larissa Greece; ^5^ Department of Radiology, Hammersmith Hospital Imperial College Healthcare NHS Trust London UK; ^6^ Department of Cardiothoracic Surgery, Hammersmith Hospital Imperial College Healthcare NHS Trust London UK

**Keywords:** CABG, heart failure, myocardial viability

## Abstract

**Background:**

We aim to provide a comprehensive review of the current state of knowledge of myocardial viability assessment in patients undergoing coronary artery bypass grafting (CABG), with a focus on the clinical markers of viability for each imaging modality. We also compare mortality between patients with viable myocardium and those without viability who undergo CABG.

**Methods:**

A systematic database search with meta‐analysis was conducted of comparative original articles (both observations and randomized controlled studies) of patients undergoing CABG with either viable or nonviable myocardium, in EMBASE, MEDLINE, Cochrane database, and Google Scholar, from inception to 2022. Imaging modalities included were dobutamine stress echocardiography (DSE), cardiac magnetic resonance (CMR), single‐photon emission computed tomography (SPECT), and positron emission tomography (PET).

**Results:**

A total of 17 studies incorporating a total of 2317 patients were included. Across all imaging modalities, the relative risk of death post‐CABG was reduced in patients with versus without viability (random‐effects model: odds ratio: 0.42; 95% confidence interval: 0.29–0.61; *p* < 0.001). Imaging for myocardial viability has significant clinical implications as it can affect the accuracy of the diagnosis, guide treatment decisions, and predict patient outcomes. Generally, based on local availability and expertise, either SPECT or DSE should be considered as the first step in evaluating viability, while PET or CMR would provide further evaluation of transmurality, perfusion metabolism, and extent of scar tissue.

**Conclusion:**

The assessment of myocardial viability is an essential component of preoperative evaluation in patients with ischemic heart disease undergoing surgical revascularization. Careful patient selection and individualized assessment of viability remain paramount.

## Graphical abstract

This graphical abstract illustrates the assessment of myocardial viability using various imaging modalities (single‐photon emission computed tomography, dobutamine stress echocardiography, cardiac magnetic resonance, positron emission tomography) for patients undergoing coronary artery bypass grafting. It details the criteria for viable and potentially viable myocardium and highlights the importance of these assessments in guiding treatment decisions and predicting patient outcomes. Key clinical considerations and evidence for myocardial viability are also presented.

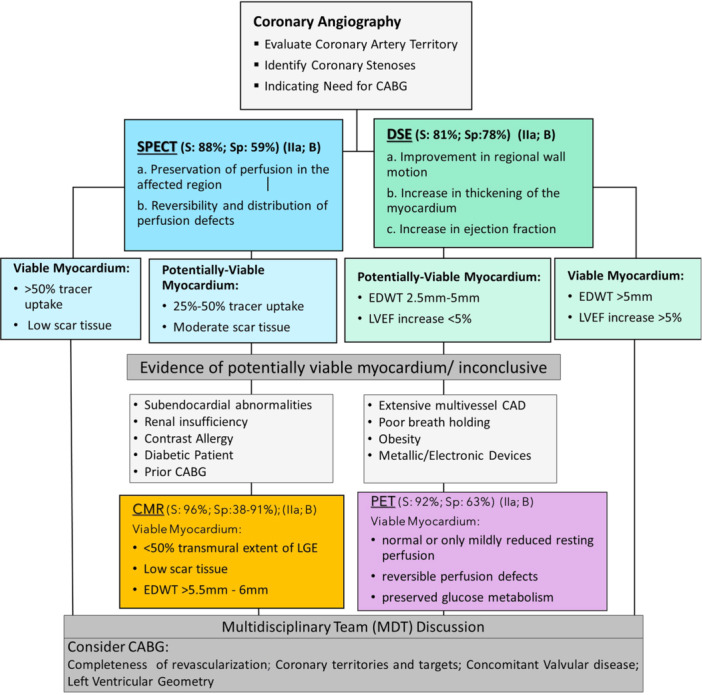

## 1 Introduction

Ischemic heart disease (IHD) remains one of the leading causes of mortality and morbidity worldwide with a rising number of patients worldwide suffering from ischemic cardiomyopathy and heart failure (HF) [1]. Ischemic cardiomyopathy, a specific form of cardiomyopathy characterized by impaired cardiac function resulting from chronic coronary artery disease and subsequent myocardial infarctions, often leads to HF. HF itself is defined as a clinical syndrome in which the heart is unable to pump sufficiently to meet the body's demands. This condition can arise from various forms of heart disease, including ischemic cardiomyopathy. Additionally, left ventricular systolic dysfunction, which involves a reduction in the ejection fraction, is a common manifestation in these patients but is not specific to any single etiology. It represents the functional consequence of past myocardial infarction events linked to coronary artery disease, the primary pathway through which ischemic cardiomyopathy causes HF.

Myocardial ischemia refers to a pathological condition where the heart muscle (myocardium) suffers due to inadequate blood supply, often necessitating percutaneous or surgical intervention to restore perfusion. Distinct from ischemia, myocardial viability denotes the presence of living heart muscle that retains the potential to recover function. This myocardium may experience ischemia if arterial blockages are present, yet not all viable myocardium undergo ischemia, and not all require revascularization [2]. Myocardial stunning is a transient and reversible impairment of myocardial function, which occurs following an episode of ischemia or reperfusion injury [2]. The underlying mechanism of myocardial stunning is thought to involve alterations in myocardial calcium handling, energy metabolism, and oxidative stress [3]. On the other hand, myocardial hibernation is a chronic and potentially reversible reduction in myocardial function and is thought to involve adaptive changes in myocardial energy metabolism, as well as structural and functional remodeling of the myocardium [4]. Hibernating myocardium can be viable or partially viable, depending on the amount of fibrosis and the stage of progression. Myocardial viability refers to the ability of the myocardium to recover function following an episode of ischemia or injury.

Coronary artery bypass grafting (CABG) represents the surgical treatment for patients with ischemic cardiomyopathy. Whilst observational studies have reported improvement in outcomes, the perioperative risk is significant, particularly in patients with poor ejection fraction who are often old, frail, and have multiple comorbidities. Although observational studies have supported the use of viability assessment, prospective studies had contradictory findings [5, 6]. Indeed, there remain many unknown elements regarding the interaction between viability, ischemia, and treatment in predicting clinical outcomes and prognosis.

Imaging for myocardial viability has significant clinical implications as it can affect the accuracy of the diagnosis, guide treatment decisions, and predict patient outcomes [2, 4]. In the past few decades, various imaging modalities have been developed to assess myocardial viability, including dobutamine stress echocardiography (DSE), cardiac magnetic resonance (CMR), single‐photon emission computed tomography (SPECT), and positron emission tomography (PET). Each of these imaging modalities studies different aspects of the pathophysiology of hibernation, producing distinctive parameters aimed at predicting functional recovery.

In this article, we focus on patients with ischemic cardiomyopathy and poor left ventricular systolic function who are undergoing CABG. These patients are at high risk for HF due to the underlying ischemic nature of their cardiomyopathy, making the assessment of myocardial viability particularly crucial for surgical planning and prognosis. The quantitative aspect of our study seeks to compare mortality between patients with viable myocardium and those without viability who undergo CABG.

## 2 Methods

### 2.1 Literature Search Strategy

A systematic review and meta‐analysis were conducted in accordance with the Cochrane Collaboration published guidelines and the Preferred Reporting Items for Systematic Reviews and Meta‐Analyses (PRISMA) statement. A literature search was conducted of EMBASE, MEDLINE, Cochrane, PubMed, and Google Scholar from inception to May 2022*.* The search terms used were: (“CMR” OR “Cardiac Magnetic Resonance” OR “Dobutamine Stress Echocardiogram” OR “DSE” OR “Stress Echocardiogram” OR “Single‐photon emission computed tomography” OR “SPECT”) AND (“coronary artery bypass grafting” OR “CABG” OR “surgical coronary revascularization”) AND (“myocardial viability” OR “cardiac viability” OR “myocardial perfusion”). Further articles were identified through the use of the “related articles” function on MEDLINE and a manual search of the reference lists of articles found through the original search. The only limits used were English language and the mentioned time frame. Patient consent and International Review Board approval were not necessary for this study as no patients were recruited.

### 2.2 Study Inclusion and Exclusion Criteria

All original articles, including both observational studies and randomized controlled trials, assessing myocardial viability through either CMR, DSE or PET/SPECT in patients undergoing CABG surgery and reporting on mortality were included. For the purpose of this study, we have clearly differentiated between “myocardial ischemia” as the condition necessitating intervention and “myocardial viability,” which refers to the functional reserve of the myocardium irrespective of the presence of ischemia. Studies were excluded from the review if: (1) inconsistencies in the data precluded valid extraction, (2) studies did not report on mortality and complications, (3) the study population did not undergo CABG, (4) the study was performed in an animal model, and (5) the size of the study population was small (<10 patients). Case reports, reviews, abstracts from meetings, and preclinical studies were excluded. By using the following criteria, two reviewers (E.T. and A.A.R.) independently selected articles for further assessment after title and abstract review. Disagreements between the two reviewers were resolved by a third independent reviewer (R.V.). Potentially eligible studies were then retrieved for full‐text assessment.

### 2.3 Data Extraction and Critical Appraisal

The data extraction and critical appraisal section is available in Supporting Information S1: File 1.

### 2.4 Data Analysis

Odds ratios (OR) with 95% confidence interval (CI) and *p* values for follow‐up mortality were calculated. Forest plots were created to represent the clinical outcomes. Chi‐squared test and *I* ^2^ test were executed for assessment of statistical heterogeneity. By using a Mantel–Haenszel random‐effects model, the OR were combined across the studies. Funnel plots were constructed to assess publication bias. All analyses were completed through the “metafor” package of R Statistical Software (version 4.0.2; Foundation for Statistical Computing, Vienna, Austria). A two‐tailed *p* value < 0.05 was considered statistically significant.

## 3 Results

### 3.1 Description of Studies and Baseline Characteristics

**Figure 1:**
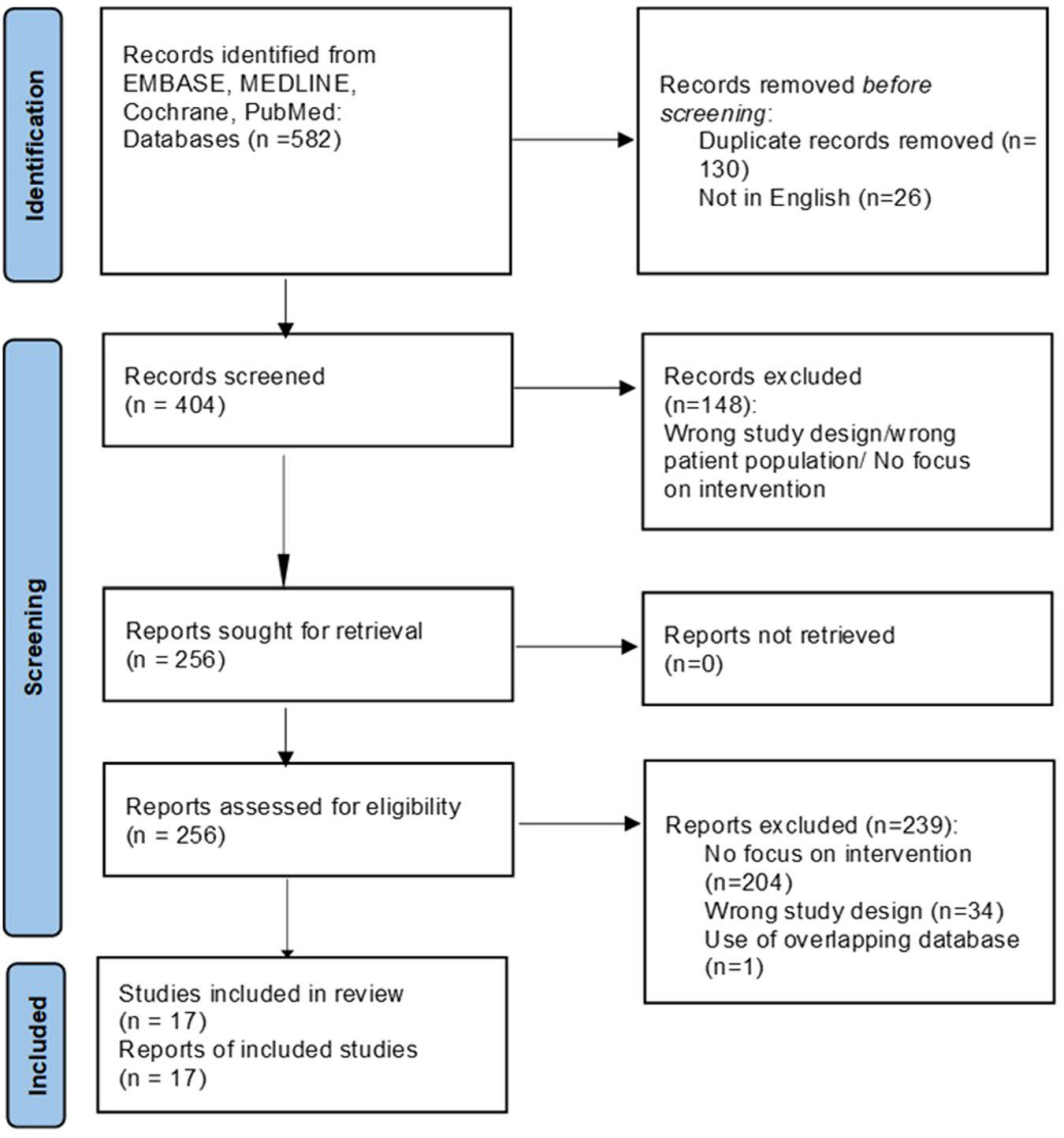
PRISMA flow chart.

The literature search identified 582 articles. Of these, 256 relevant articles were read in full and assessed according to our inclusion and exclusion criteria. Following critical appraisal, a total of 17 studies [7, 8, 9, 10, 11, 12, 13, 14, 15, 16, 17, 18, 19, 20, 21, 22, 23] incorporating a total of 2317 patients were included (Table 1 and Figure 1). The studies described outcomes of patients with HF undergoing CABG and being assessed through either CMR, DSE, SPECT or PET modality for myocardial viability. Figure S1 illustrates the study selection process. Five studies included were retrospective, while the remaining were prospective in nature. Table 1 reports the baseline characteristics of the included patients. Table 2 reports the markers of viability per imaging modality. Supporting Information S1: Table 4 reports the sensitivity, specificity, NPV, and PPV for each modality.

**Table 1:** Baseline characteristics of all studies included.

Study	Year	Design	Imaging	Population	Males, *n* (%)	Age	Follow‐up (months)
Gerber et al.	2012	Prospective	CMR	144	130 (87.0)	66 ± 10	36
Lee et al.	2016	Retrospective	CMR	146	105 (72.0)	64 ± 9.0	113 (97–122)
Kancharla et al.	2016	Retrospective	CMR	185	133 (71.9)	63.2 ± 11.5	97 (84–100)
Yap et al.	2020	Retrospective	CMR	79	73 (92.4)	57.5 ± 8.5	47 ± 32
Hwang et al.	2017	Retrospective	CMR	77	63 (81.8)	64 ± 11	11 (3–12)
Maruskova et al.	2009	Prospective	CMR	79	64 (81.0)	65 ± 9.0	29 (6–53)
Li et al.	2017	Prospective	SPECT, PET	115	94 (81.7)	67.2 ± 10.1	35 ± 13
Liu et al.	2018	Retrospective	PET	53	67 (82.0)	54.8 ± 12.5	32
Panza et al.	2019	Prospective	SPECT, DSE	298	521 (87)	60.7 ± 9.4	125
Cao et al.	2020	Prospective	PET	118	—	59 ± 8.0	30 ± 12
Meluzin et al.	2003	Prospective	DSE	124	119 (96.0)	57 ± 9.0	27 ± 23
Meluzin et al.	2003	Prospective	DSE	227	207 (91.2)	59 ± 9.0	30 ± 12
Meluzin et al.	1998	Prospective	DSE	133	129 (97.0)	58.0 ± 8.0	20 ± 12
Sicari et al.	2003	Prospective	DSE	188	162 (86.2)	61 ± 10.0	37
Sicari et al.	2001	Prospective	DSE	124	100 (80.6)	60 ± 10.10	38
Acampa et al.	2004	Prospective	SPECT, DSE	253	228 (90.0)	53 ± 12	52 ± 29

**Table 2:** Clinical markers of viability per imaging modality.

	Clinical marker of viability	Effect
SPECT	Percentage of peak tracer uptake	This is the most commonly used criterion to identify viable myocardium A cutoff of >50% tracer activity is used to identify viable myocardium
	End‐diastolic wall thickness	EDWT of >5 mm indicates viability
	Extent of myocardial scar tissue	Patients with a small amount of scar tissue are more likely to have viable myocardium and better outcomes following CABG
	Distribution of perfusion defects	Patients with reversible perfusion defects in the anterior or lateral walls of the left ventricle are more likely to have viable myocardium and better outcomes following CABG
	Contractile reserve	Patients who show improvement in wall motion during stress testing have a higher likelihood of having viable myocardium and better outcomes following CABG; >5 viable segments indicates the threshold for improved outcomes
DSE	Wall motion score index (WMSI):	A lower WMSI indicates better contractile reserve in the myocardial tissue; thus, revascularization is more likely to improve cardiac function and overall outcomes
	End‐systolic volume (ESV)	A decrease in ESV after dobutamine infusion indicates viability. A decrease of more than 10% is considered significant
	End‐diastolic volume (EDV)	An increase of more than 10% is considered significant
	Ejection fraction (EF)	An increase in EF after dobutamine infusion indicates viability. An increase of more than 5% is considered significant
	Contractile reserve	An improvement in contractile reserve after dobutamine infusion indicates viability
PET	Resting perfusion:	The presence of normal or only mildly reduced resting perfusion in an area of the myocardium is a strong indicator of viable tissue
	Reversible perfusion defects	The presence of reversible perfusion defects on PET imaging indicates the presence of viable myocardium that is ischemic and may benefit from revascularization
	Glucose metabolism	The presence of preserved glucose metabolism in an area of the myocardium suggests the presence of viable tissue
	Contractile function	The presence of preserved or only mildly impaired contractile function in an area of the myocardium is a strong indicator of viable tissue
	Wall thickness	The presence of preserved or only mildly reduced wall thickness in an area of the myocardium suggests the presence of viable tissue
CMR	Late gadolinium enhancement (LGE):	The presence of LGE on CMR imaging indicates the presence of nonviable tissue The threshold for viability is typically set at 50% transmural extent of LGE. Areas of the myocardium with more than 50% LGE are considered nonviable and unlikely to benefit from revascularization
	EDWT	EDWT > 5.5 to 6 mm is considered indicative of viable myocardium and improved outcomes following revascularization
	Regional wall motion abnormalities:	The threshold for viability is typically set at the presence of mild to moderate wall motion abnormalities, indicating that the tissue may be viable and may benefit from revascularization
	Transmural extent of scar	Areas of the myocardium with only subendocardial scar have a higher chance of functional recovery after revascularization than areas with transmural scar The threshold for viability is typically set at less than 50% transmural extent of scar, indicating that the tissue may be viable and may benefit from revascularization
	T2‐weighted imaging	CMR can detect areas of edema and inflammation, which are indicative of acute myocardial injury The threshold for viability on T2‐weighted imaging is not well established, but areas of edema and inflammation are generally considered to be viable and may benefit from revascularization
	Dobutamine stress CMR	Dobutamine stress CMR can be used to assess contractile reserve in areas of the myocardium that are thought to be viable The presence of contractile reserve is indicative of viable tissue that may benefit from revascularization

### 3.2 Markers of Myocardial Viability

#### 3.2.1 Markers of Myocardial Viability Across Imaging Modalities

Markers of myocardial viability were assessed using four key imaging modalities, summarized in Supporting Information S1: Tables 1–3 and Table 2.

#### 3.2.2 Mortality and Myocardial Viability

**Figure 2:**
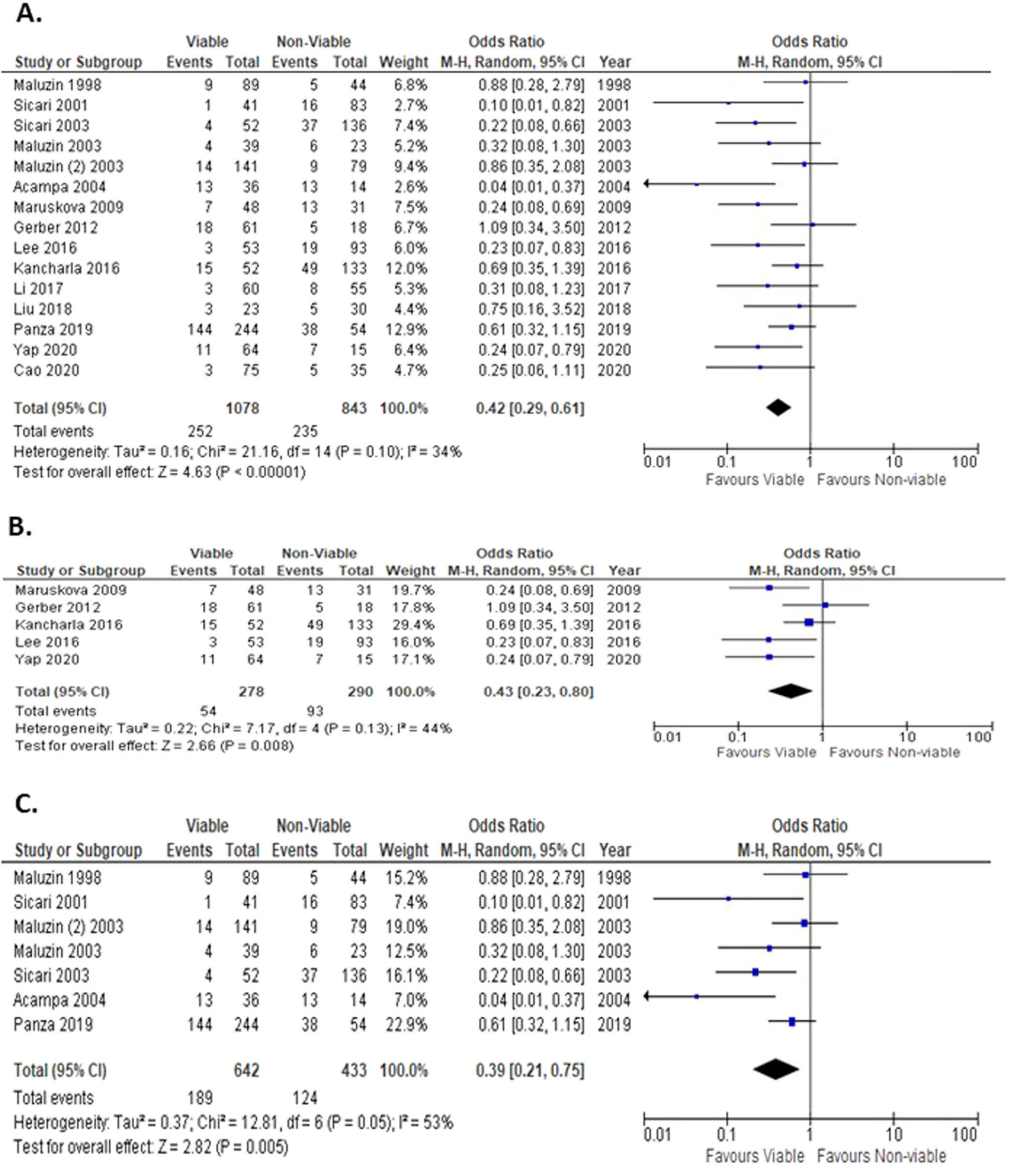
Forest plot for all mortality across all imaging modalities (A), across CMR (B) and DSE (C).

Patients with viable myocardium were compared to those with nonviable myocardium across all imaging modalities, with 15 studies reporting on mortality outcomes postoperatively (Figure 2). The overall OR for mortality showed a statistically significant difference in favor of patients with viable myocardium (random‐effects model: OR: 0.42; 95% CI: 0.29–0.61; *p* < 0.001). There was evidence of low heterogeneity among studies reporting on mortality. When CMR and DSE were analyzed separately, the statistically significant difference in favor of patients with viable myocardium was maintained.

## 4 Discussion

This meta‐analysis of 17 studies, incorporating a total of 2317 patients, revealed that the relative risk of death after CABG is lower in patients with viable myocardium as opposed to those with nonviable myocardium. While our study emphasizes the role of assessing myocardial viability in surgical decision‐making and prognosis, it is crucial to differentiate this from myocardial ischemia, which independently influences the urgency and type of intervention required. Our results demonstrate that regardless of viability, the presence of ischemic myocardium correlates with improved cardiovascular outcomes post‐CABG, highlighting the importance of addressing both conditions distinctly. Our findings resonate with the conclusions drawn by Liga et al. [24] regarding the challenges in myocardial viability assessment for ischemic cardiomyopathy. Both studies acknowledge the complexity of determining the optimal treatment path and the limitations of current methodologies. However, while Liga et al. suggest a cautious approach due to mixed outcomes from myocardial viability assessments, our review goes a step further by proposing a strategic use of various imaging modalities, such as DSE, CMR, SPECT, and PET. We specifically address how each modality can be optimally employed to refine myocardial viability assessments, enhancing their predictive power in guiding surgical decisions. This tailored approach aims to maximize clinical outcomes by aligning the chosen imaging technique more closely with individual patient characteristics and clinical needs.

### 4.1 Optimal Use of Imaging for Viability

Due to the nascent stage of this research/clinical area, clear and definitive guidelines have yet to be established. This dynamic landscape underscores the importance of continuous exploration and validation to ensure the best patient care. The 2021 AHA/ACC/ASE/CHEST/SAEM/SCCT/SCMR Guideline for the Evaluation and Diagnosis of Chest Pain recommends the use of CT angiography or stress imaging for intermediate‐ to high‐risk patients with stable chest pain and no known CAD [25]. The stress imaging modalities mentioned include DSE, PET, SPECT, and CMR. The 2019 ESC Guidelines for the diagnosis and management of chronic coronary syndromes only recommend performing CMR in patients with an inconclusive echocardiographic test [26]. Our algorithm represents the first attempt to provide some foundations to inform clinicians on how to navigate these imaging modalities.

### 4.2 DSE

Echocardiography is useful for determining left ventricular ejection fraction (LVEF), detecting wall motion abnormalities, and assessing wall thickness and LV size [13, 18]. An end‐diastolic thickness at rest below 5 mm, accompanied by akinesia or dyskinesia, indicates nonviable myocardium [13, 18]. A myocardial wall thickness over 5 mm in end‐diastole indicates viable myocardium with a high sensitivity but low specificity [27]. A severely dilated LV indicates nonviable myocardium, and an LV end‐systolic volume >130 mL predicts a higher rate of cardiac events after revascularization, even with viable myocardium [28].

DSE can predict functional recovery after revascularization, even in cases without contractile reserve [16, 18]. Revascularization may not lead to LV contractility recovery in cases of low myocardial viability, but it could help avoid LV remodeling [29]. A contractile reserve in at least five segments on DSE is a good predictor of LV function and clinical outcomes [30]. A characteristic response of hibernating myocardium is the biphasic response, constituted by an initial improvement in contractility indicating viability, followed by a later worsening due to ischemia [31, 32]. This has been linked to an up to 75% improvement in the regional ventricular improvement of patients with IHD undergoing CABG [31]. Another characteristic response of hibernating myocardium to dobutamine is the worsening of contractile function as the dose is increased [28, 30].

### 4.3 SPECT

SPECT imaging is used to evaluate the perfusion and metabolism of the myocardium. The presence of viable myocardium is determined by the detection of reversible perfusion defects, preserved metabolism, and contractile reserve [9, 18, 33]. SPECT is a method used to measure regional tracer concentration in the myocardium by using radionuclide‐labeled tracer. This measurement can indicate viability by calculating the percentage of peak uptake of the tracer. The viability assessment can be conducted using rest images only or with a stress/rest testing protocol [34]. The radiotracers sequester within myocytes that have intact cell membranes. As a result, SPECT cannot assess the transmural extent of variability within the left ventricular wall, and myocardial viability is interpreted as an all‐or‐none phenomenon [34].

### 4.4 DSE and SPECT as the First Step of Viability Assessment

**Figure 3:**
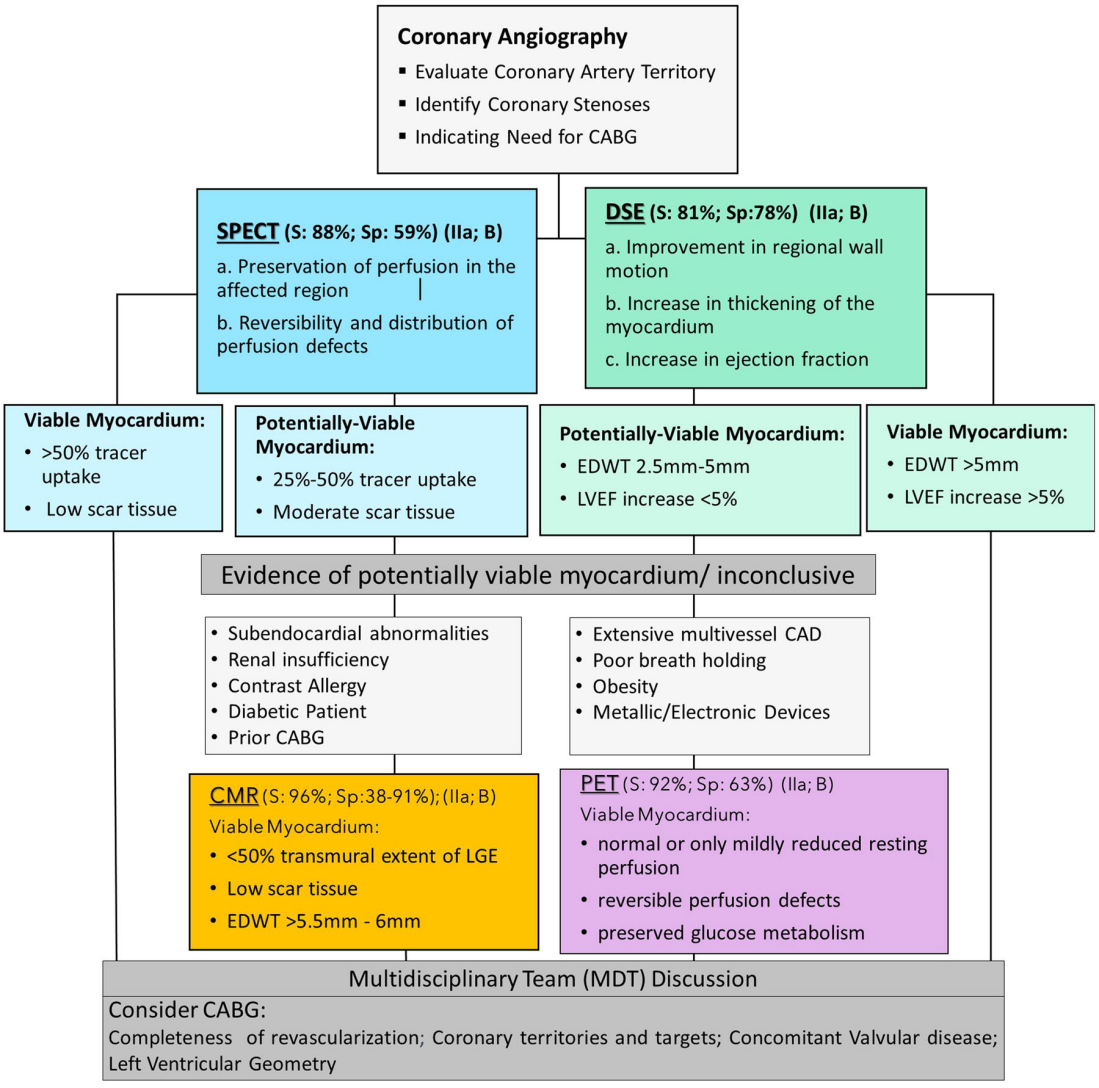
Proposed algorithm for myocardial viability assessment of patients with IHD being considered for CABG.

SPECT > DSE (Figure 3): SPECT may be preferred in patients with severely limited exercise capacity or extensive, diffuse areas of myocardial scar tissue. SPECT provides a more comprehensive assessment of myocardial perfusion and metabolism throughout the entire myocardium, making it more useful in identifying the overall extent of viable myocardium in these patients [18]. Additionally, SPECT may be preferred over DSE in patients with significant valvular heart disease.

DSE > SPECT: DSE may be preferred in patients who are unable to undergo nuclear imaging due to contraindications such as renal impairment or iodine allergy. Second, DSE may be preferred in patients with left bundle branch block (LBBB). LBBB can cause false‐positive results that can lead to inaccurate assessment of myocardial viability [35]. DSE also allows for the assessment of regional wall motion abnormalities, which may be a more sensitive marker of myocardial viability in patients with single‐vessel disease or small infarcts where the area of viable myocardium is small, and the spatial resolution of SPECT may not be adequate to detect viability.

### 4.5 PET

PET offers superior spatial resolution (Figure S1) compared to SPECT, and rest perfusion can be evaluated using various tracers [36]. Hibernating myocardium can be identified by assessing glucose uptake in the myocardium. In the fasted state, ischemic myocardium primarily takes up FDG, while scar tissue and normal myocardium do not [36]. However, one limitation of PET is the inconsistency in FDG uptake, which can be influenced by factors such as cardiac output, HF, ischemia, and sympathetic activity.

### 4.6 CMR

CMR can detect global LV function and regional wall motion and assess myocardial viability using LV end‐diastolic wall thickness (EDWT) or dobutamine stress testing [7, 8]. It is particularly useful in patients with a poor ultrasound window, allowing for precise evaluation of wall thickness and contractile function. Gadolinium‐based contrast agents can detect cellular damage and impaired microperfusion, and late gadolinium enhancement (LGE) can produce hyperenhanced areas in nonviable myocardium due to the retained contrast. The extent and the transmural extension of the myocardial scar can be accurately quantified due to superior spatial resolution. If transmurality on the LGE of a myocardial segment is >50%, it is considered nonviable myocardium, while if it is δ 50%, it is viable and correlates with LV segmental contractile restoration after revascularization [37]. CMR allows for evaluation of the contractile reserve in instances in which LGE is <50% and, with a thickening of >2 mm in segments, which are akinetic or dyskinetic, indicating viability [38]. Generally, an EDWT higher than 5.5–6 mm is considered indicative of viable myocardium and improved outcomes following revascularization [39]. Nevertheless, it must be noted that around 20% of thinner and lower contractility segments will still exhibit viability and an improvement in contractility following revascularization [39].

### 4.7 PET and CMR as the Second Step

CMR > PET (Figure 2): CMR has superior spatial resolution compared to PET, making it better at detecting subendocardial abnormalities [40]. CMR may be preferred in diabetic patients undergoing CABG and patients with renal insufficiency, as they may have difficulties with insulin regimens or are at risk of nephrotoxicity with contrast agents used in PET. However, CMR may not accurately identify viable myocardium in patients with prior CABG due to the presence of scar tissue and surgical grafts [40]. Nephrogenic systemic fibrosis is also a rare complication of gadolinium in patients with renal impairment.

PET > CMR: PET may be preferred in patients with multivessel disease as it can evaluate myocardial viability in all coronary territories simultaneously, whereas CMR may require multiple imaging sessions. PET may also be better at identifying hibernating myocardium (as it can evaluate myocardial perfusion and metabolism simultaneously) and can be performed safely in patients with metallic implants or devices [40]. In cases where CMR produces images of suboptimal quality motion artifacts or poor breath‐holding, PET may be preferred. PET may be more practical in regions or institutions where it is more readily available than CMR.

### 4.8 The Benefit of Myocardial Viability Assessment for Surgical Revascularization

#### 4.8.1 Ventricular Function Recovery

Traditionally, an improvement in function after revascularization has been considered the final proof of viability. From a clinical point of view, improvement in global LV function has been demonstrated to be a more powerful predictor of prognosis than improvement in regional function [41]. However, most viability assessment studies have only evaluated segmental improvement and the exact proportion of viable segments needed to result in improvement in LVEF after revascularization is unclear. Some studies [13, 14, 15] suggest that the presence of ≥6 dysfunctional but viable segments identifies patients with the best prognosis, while others [18] suggest that ≥5 viable segments (>38% of total myocardium) is the best cut‐off. Scar tissue on LGE‐CMR has also been proposed to be superior to viable myocardium in predicting cardiac functional recovery in patients undergoing CABG [22]. The number of scar segments was found to be the best independent negative predictor of global functional recovery, with high sensitivity and specificity. Therefore, the analysis should consider both viable and scar segments.

Nevertheless, the improvement of LV function is not the only and most important mechanism for improved outcomes after CABG. Multiple factors are involved, including transmurality of the viable areas, regional nature of LV dysfunction, completeness and durability of revascularization, and the degree of perioperative myocardial damage [6]. The most important mechanism for CABG is protection from future acute coronary events and preventing further damage and sudden death [6]. Furthermore, CABG improves exercise tolerance and quality of life, as well as survival. In patients with ischemic LV dysfunction, sudden arrhythmic or ischemic deaths are not correlated with LV ejection fraction [42]. The risk of death due to ventricular arrhythmic events and myocardial infarction can be influenced by revascularization, independent of any effect on contractile function [42]. In fact, reduction in LV volume and sphericity after revascularization are important prognostic factors outside the improvement of regional wall thickening and LV ejection fraction [43].

#### 4.8.2 Viability and Mortality

Our analysis suggests that the relative risk of mortality of patients undergoing surgical revascularization with myocardial viability was reduced when compared to those without viability. Medical therapy with surgical revascularization in patients with HF who had a previous MI has shown improved survival and long‐term prognosis, compared to medical therapy alone [44]. Previous studies agree with the assessment of viability before CABG in patients with viable, dysfunctional myocardium [34]. Nevertheless, it must be noted that the definition of viability as a dichotomous variable is intrinsically flawed, as myocardial hibernation is thought to be a spectrum from the time of injury. Due to the low granularity of the data with regard to mortality and viability, an in‐depth analysis was not deemed feasible.

The STICH trial [6] did not provide substantial support for the notion that the assessment of myocardial viability significantly influences the long‐term benefits of surgical revascularization in patients with ischemic cardiomyopathy. Although the study revealed a lower incidence of all‐cause mortality with CABG plus medical therapy compared to medical therapy alone, the interactions between myocardial viability and CABG treatment effect were not statistically significant for any of the endpoints [6]. However, several limitations of the study need to be addressed. First, the use of LGE for myocardial viability assessment was not widespread at the inception of STICH, unlike with DSE and SPECT. Second, only 49% of patients enrolled were tested for viability, and out of those only 19% had nonviable myocardium, potentially leading to an underpowered assessment. Third, patients with viable myocardium noted a significant increase in LVEF.

Additionally, two prospective randomized trials—the Heart Failure Revascularization (HEART) trial [45], the PET And Recovery following Revascularization (PARR‐2) trial [46]—have explored this concept. The HEART trial found no additional benefit from revascularization even in the presence of viable myocardium, but the trial was stopped early due to poor recruitment. The PARR‐2 trial found a significant benefit for their primary outcome only when PET recommendations, where followed, potentially supporting the concept of viability testing.

### 4.9 Optimal Patient Selection

#### 4.9.1 Coronary Angiography: Coronary Artery Targets and Territories

Coronary angiography plays a crucial role in the preoperative evaluation of patients with suspected myocardial ischemia considered for revascularization procedures. When myocardial viability assessment is taken into consideration in the context of revascularization, regardless of the degree of viability, careful attention should be paid to the feasibility of surgical revascularization. In fact, anatomical correspondence between the viable segment and the involved coronary artery should be evaluated using coronary angiography [47].

A viability study in conjunction with coronary angiography can justify when not to revascularize a nonviable territory with poor anastomotic targets. Evidence of viability in the LAD territory warrants the use of a left internal mammary artery‐to‐LAD graft for optimal benefits and provides a justification against the risks of CABG, especially in patients with longer life expectancy. A study [48] using thallium‐201 SPECT perfusion imaging indicated that in patients with severe LV dysfunction (LVEF ≤ 35%), myocardial viability in the LAD territory predicts a significant postoperative improvement in functional class and LVEF of at least 10% or more after CABG with severe LV dysfunction. Yang et al. [22] agree that the number of scar segments, total scar score, and the number of scar segments in LAD territory correlated very well with LVEF after CABG. Thus, the final clinical outcome should be multifactorial, considering the viable segment in conjunction with adequate coronary targets and territories.

#### 4.9.2 LV Geometry and Viability

Assessing LV geometry is crucial in selecting patients for revascularization therapy [49]. Several factors, including anterior infarct location, infarct size, and patency of the infarct‐related artery, are associated with progressive LV dilatation [50]. LV remodeling, which involves changes in LV geometry and function, is primarily caused by transmural myocardial necrosis [50].

A key determinant of LV function is an increased LV volume, which is associated with reduced LV ejection fraction, increased risk of HF, and mortality. The shape of the LV is also important, with a more elliptical shape associated with better outcomes and a spherical shape with poorer outcomes [49]. Decreased LV wall thickness can result in impaired LV contractility and increased risk of HF. Other geometrical factors, such as LV mass, end‐systolic and end‐diastolic wall stress, and the presence of LV aneurysm, can also impact LV function and patient outcomes [50, 51].

The presence of viable myocardium in the outer layers of the ventricular wall is associated with the maintenance of LV size, and revascularization therapy may help prevent further LV dilation and dysfunction in these patients [52]. However, in patients with extensive myocardial damage, the absence of viable myocardium may lead to further LV dilation and dysfunction, and revascularization therapy may not be beneficial [52].

### 4.10 Complete vs. Incomplete Revascularization: The Importance of Distinguishing CABG from PCI

The benefits of surgical revascularization extend far beyond standard morbidity/no patients come. Indeed, as demonstrated by data obtained from the STICH trial and ISCHEMIA trial, protection against fatal myocardial infarction and future coronary events leading to sudden death has been arguably outlined as crucial advantages provided by CABG [6, 44]. Furthermore, surgical coronary revascularization might be able to contribute to both the functioning and electrical stability of cardiac myocytes embedded in scar tissue and not directly contribute to the left ventricular systolic function. An important point to make is that the mechanism of revascularization significantly differs between percutaneous coronary intervention (PCI) and CABG. PCI is only focused on flow‐limiting lesions and is mostly unable to limit new infarcts, which tend to arise in nonflow–limiting stenosis. CABG on the other side provides flow distally to the occlusion, provides surgical “collateralization,” and has been shown to cause infarct reduction [53]. Interestingly, the REVIVED‐BCIS2 trial showed that for patients experiencing severe ischemic left ventricular systolic dysfunction and undergoing optimal medical therapy, revascularization through PCI did not lead to a reduced occurrence of all‐cause mortality or hospitalization due to HF [54]. Therefore, future trials should not consider “any” revascularization strategy as equivalent, especially in the context of viability.

## 5 Conclusions

Surgical revascularization appears to reduce the relative risk of death of patients with substantial amounts of viable myocardium. Although the optimal adoption of myocardial viability imaging for revascularization remains an area of controversy, it represents a powerful tool for the preoperative evaluation of patients with moderate–severe ventricular dysfunction. Generally, based on the local availability and expertise, either SPECT or DSE should be considered as the first step of viability assessment. In the presence of potentially viable myocardium PET or CMR would provide further evaluation of the transmurality, perfusion metabolism, and extent of scar tissue. Nevertheless, multiple factors are involved in the improvement of CABG outcomes in the context of viability, including transmurality of the viable areas, regional nature of LV dysfunction, completeness, and durability of revascularization, and the degree of perioperative myocardial injury.

## Conflicts of Interest

The authors declare no conflicts of interest.

## Supporting information

Supporting information.

Supporting information.

## Data Availability

The data that support the findings of this study are available from the corresponding author upon reasonable request.
