# Supplementary material for: Assessment of Myocardial Viability in Ischemic Cardiomyopathy With Reduced Left Ventricular Function Undergoing Coronary Artery Bypass Grafting
Source: Clin Cardiol. 2024 Jul 2;47(7):e24307. doi: 10.1002/clc.24307 (PMC11217808; doi:10.1002/clc.24307)
Supplement: Supplementary file 1 — Supporting information. [file CLC-47-e24307-s001.docx]

**Myocardial viability in heart failure patients undergoing coronary artery bypass grafting: a systematic review and meta-analysis.**

**(Running head: Myocardial Viability Imaging for CABG)**

Arian Arjomandi Rad^1*^ MBBS, BSc; Eleni Tserioti^2*^ BSc, Dimitrios E. Magouliotis^3^ MD, PhD; Robert Vardanyan^2^ MBBS, BSc; Ilias V. Samiotis^3^ MD, PhD; John Skoularigis^4^ MD; Ben Ariff^5^ MD, PhD; Andreas Xanthopoulos^4^ MD, PhD; Filippos Triposkiadis^4^ MD; Roberto Casula^2,6^ MD; Thanos Athanasiou^2,3,6^ MD, PhD


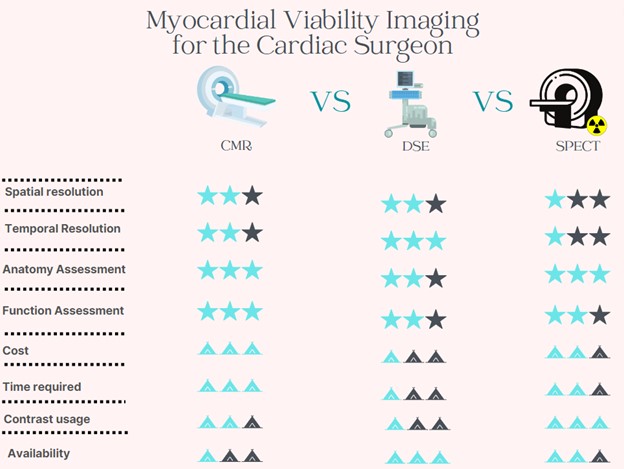


**Supplementary Figure 1: simplified diagram comparing the three different imaging modalities for myocardial viability assessment.**

**Data Extraction and critical appraisal**

All full texts of retrieved articles were read and reviewed by two authors (E.T and A.AR) and the inclusion or exclusion of studies was decided unanimously. When there was disagreement, a third reviewer (R.V.) made the final decision. Using a pre-established protocol, the following data were extracted: first author, study type and characteristics, number of patients, population demographics, imaging modality, Median follow-up, average late-gadolinium enhancement (LGE) score, wall motion score/ abnormality, mean number of dysfunctional but viable segments, minimum number of prognostic viable segments and main study findings. For this review, a data extraction sheet was developed, and pilot-tested on 3 randomly selected included studies, whereupon the sheet was refined accordingly. Data extraction was performed by 2 review authors (E.T and A.AR). A third author (R.V.) validated the correctness of the tabulated data. Potential inter-reviewer disagreements were resolved by consensus. The primary outcome was follow-up mortality.

**Strengths and Limitations of the Review:**

**Strengths:**

1. **Comprehensive Scope:** This review meticulously analyzes a wide range of imaging modalities used to assess myocardial viability in patients undergoing coronary artery bypass grafting (CABG), providing a holistic overview of the current state of knowledge.
2. **Methodological Rigor:** Adhering to PRISMA guidelines, the systematic review ensures a high standard of transparency and reproducibility. The inclusion of multiple databases and rigorous selection criteria enhance the breadth and quality of the literature assessed.
3. **Clinical Relevance:** By linking specific imaging modalities to patient outcomes, this review directly contributes to enhancing clinical decision-making, offering practical insights that can be applied in clinical settings to improve patient care.

**Limitations:**

1. **Variability in Study Designs:** The included studies vary in design, sample size, and methodology, which might introduce heterogeneity that can affect the generalizability of the findings.
2. **Lack of Prospective Validation:** While the review synthesizes existing studies, the recommendations proposed for the use of various imaging modalities lack direct prospective validation in clinical practice, which is essential for confirming their effectiveness.
3. **Potential Bias in Study Selection:** Despite rigorous methods, selection bias may still influence the outcomes, particularly due to the exclusion of non-English language studies or unpublished data, which could offer additional insights.

**Implications for Future Research:**

This study’s findings on myocardial viability assessment and the stratification of imaging modalities provide several avenues for future research:

Prospective Validation: Future studies should focus on prospectively validating the clinical algorithms proposed in this review. Such studies could evaluate the effectiveness and accuracy of different imaging modalities in predicting post-CABG outcomes across diverse patient populations.

Integration with Other Biomarkers: Integrating imaging findings with molecular and genetic biomarkers may enhance the precision of myocardial viability assessments. Research into multimodal strategies could provide a more comprehensive understanding of patient prognosis and treatment optimization.

Cost-Effectiveness Analysis: Given the varying costs and availability of imaging technologies like DSE, CMR, SPECT, and PET, future research should also address the cost-effectiveness of these modalities in different healthcare settings. This would aid in making informed decisions about resource allocation in healthcare systems globally.

Long-term Outcomes: There is a need for longitudinal studies to track the long-term outcomes of patients categorized by myocardial viability, particularly those undergoing CABG. Such studies could shed light on the long-term benefits and potential risks associated with specific treatment decisions based on viability assessment.

Technological Advancements: As imaging technology evolves, future research should continuously evaluate the impact of newer imaging techniques on the accuracy of myocardial viability assessments. This could include advancements in artificial intelligence and machine learning algorithms to interpret imaging data

Global Application: Considering the variability in medical infrastructure and patient demographics across different regions, international multicenter studies could evaluate the applicability of the findings globally, ensuring that the conclusions drawn are robust and widely applicable.

Suppl.Table 1: **Studies included using CMR**

| Study | Baseline LVEF (%) | Mean n of dysfunctional viable segments | Mean LGE Score | Average Transmurability/number of transmural segments | Wall motion score/abnormality | Minimum n of non-viable segments for prognostic value |
| --- | --- | --- | --- | --- | --- | --- |
| Gerber et al. | ≤35% | 8±5 | **-** | **15 ± 11** | **WMS:** 36±5 | ≥4 non-viable segments |
| [Lee](https://onlinelibrary.wiley.com/action/doSearch?ContribAuthorRaw=Meluz%C3%ADn%2C+Jaroslav) et al. | <50% | 3.8 ± 1.7 | **7.2±8.2** | **-** | **WMA:** 5.1±4.8 | LVEF<50% prognostic |
| [Kancharla](https://onlinelibrary.wiley.com/action/doSearch?ContribAuthorRaw=Meluz%C3%ADn%2C+Jaroslav) et al. | 38% | 2±2 | **4±3** | **2±2** | - | ≥4 non-viable segments |
| [Yap](https://onlinelibrary.wiley.com/action/doSearch?ContribAuthorRaw=Meluz%C3%ADn%2C+Jaroslav) et al. | ≤35% | 3±3.1 | **Cutoff of 50% LGE best threshold for segmental viability** | **-** | **WMSI:** 2.5±0.6 | ≥4 non-viable segments |
| Yang et al. | 33% | -  Dysfuctional:12.8±3.6  Scar: 3.6±2 | **Transmural extent LGE**<**50% =viable segment** | **-** | **WMS:** 18.3±6.1 | ≥4 non-viable segments |
| Hwang et al. | 29% | 9±4 | **Transmural extent LGE**<**50% =viable segment** | - | WMS: 31.7 ± 5.9 | ≥2 non-viable segments |
| Maruskowa et al. | 30% | 5.2±3.4 | **-** | **-** | - | < 5 dysfunctional but viable segments |

Suppl.Table **2:** Studies included using DSE

| Study | Modality | Inclusion criteria | Mean n of dysfunctional but viable segments | Optimal cutoff value of myocardial viability to show benefit of CABG* | Left ventricular function  Improvement  (before and after revasc) |
| --- | --- | --- | --- | --- | --- |
| Panza, J. et al. | SPECT  DSE | LVEF≤35% | SPECT: ≤11 viable segments  DSE: ≤5 segments | **no significant difference** between the presence or absence of myocardial viability with a cut-off ≥4 or ≥2 segments | - |
| Meluzin et al. | DSE | LVEF≤30% | 3.8 ± 1.7 | **≥2** dysfunctional but viable | LVEF>7% |
| [Meluzín et al.](https://onlinelibrary.wiley.com/action/doSearch?ContribAuthorRaw=Meluz%C3%ADn%2C+Jaroslav) | DSE | LVEF≤30% | - | **≥6** segments dysfunctional but viable | LVEF>10% |
| [Meluzín et al.](https://onlinelibrary.wiley.com/action/doSearch?ContribAuthorRaw=Meluz%C3%ADn%2C+Jaroslav) | DSE | LVEF≤40% | 7.4 ± 1.7 | **≥6 segments** dysfunctional but viable | LVEF & WMSI * |
| [Sicari, R. et al.](https://pubmed.ncbi.nlm.nih.gov/?term=Sicari+R&cauthor_id=14636900) | DSE | LVEF≤35% | - | improvement of **≥0.40** in WMSI | NA |
| [Sicari, R. et al.](https://pubmed.ncbi.nlm.nih.gov/?term=Sicari+R&cauthor_id=14636900) | DSE | LVEF≤35% | - | improvement of ≥0.20 in WMSI | NA |
| [Acampa, W. et al.](https://link.springer.com/article/10.1007/s00259-004-1693-8#auth-Wanda-Acampa) | DSE | -  Mean LVEF 38% | Pts with events:  5.5 ± 2.3  No events: 4.6 ± 2.6  (viable segms) | **≥5 segments** dysfunctional but viable | LVEF ≥5% |

Suppl.Table **3: Studies included using PET/SPECT**

|  | Baseline LVEF (%) | | Baseline LVEDVI (ml/m^2^) | |  |
| --- | --- | --- | --- | --- | --- |
| Study | Low viability LMV` | High Viability HMV | Low Viability LMV | High Viability HMV | LV ejection improvement by ≥5% |
| Li et al. | 35.7 ± 5.1 | 38.3 ±3.8 | LVDV (ml) 210.8 ±46.9 | LVDD (ml) 186.6±42.7 | ≥7 viable segments (≥41% of total myocardium)- |
| [Panza](https://onlinelibrary.wiley.com/action/doSearch?ContribAuthorRaw=Meluz%C3%ADn%2C+Jaroslav) et al. | 23.3±9.1 | 27.0±8.2 | 140±53.8 | 116±35.1 | ≥11 viable segments |
| Cao et al. | 34.2±3.5 | 33.5±3.8 | 118±35 | 114±31 | A ratio of viable to total myocardium in the LV≥10% |
| Acampa et al. | 37±8 | 39±6 | - | - | ≥5 viable segments (38% of total myocardium) |
| [Liu](https://onlinelibrary.wiley.com/action/doSearch?ContribAuthorRaw=Meluz%C3%ADn%2C+Jaroslav) et al. | Scar burden ≥10%  7.8±10.6 | Scar burden <10%  9.56±9.7 | Scar burden ≥10%  -5.3±6.3 | Scar burden <10%  -6.5±5.5 | Functional recovery following surgical revascularization not associated with mortality benefit |

**Supplemental Table 4: Sensitivity, Specificity, NPV, PPV of the cardiac imaging techniques for myocardial vialibility**

| Investigation | Sensitivity (%) segments | Specificity (%) segments | PPV (%) segments | NVV (%) segments |
| --- | --- | --- | --- | --- |
| **CMR**[43] |  | | | |
| Delayed enhancement | 95 | 51 | 69 | 90 |
| Dobutamine stress | 81 | 91 | 93 | 75 |
| End-diastolic wall thickness | 96 | 38 | 71 | 85 |
| **DSE** [44] | 81 | 78 | 75 | 83 |
| **PET**[45] | 92 | 63 | 74 | 87 |
| **SPECT (Th 201)**[46] | 88 | 59 | 69 | 80 |

*References:* [43] CMR Imaging Assessing Viability in Patients With Chronic Ventricular Dysfunction Due to Coronary Artery Disease: A Meta-Analysis of Prospective Trials | JACC: Cardiovascular Imaging n.d.https://www.jacc.org/doi/10.1016/j.jcmg.2012.02.009 (accessed December 19, 2022).

[44] Schinkel AFL,Bax JJ,Poldermans D,Elhendy A,Ferrari R,Rahimtoola SH.Hibernating myocardium: diagnosis and patient outcomes.Curr Probl Cardiol. 2007;32:375–410.

[45] MacHac J.Cardiac positron emission tomography imaging.Semin Nucl Med. 2005;35:17–36.

[46] Bax JJ,Wijns W,Cornel JH,Visser FC,Boersma E,Fioretti PM.Accuracy of Currently Available Techniques for Prediction of Functional Recovery After Revascularization in Patients With Left Ventricular Dysfunction Due to Chronic Coronary Artery Disease: Comparison of Pooled Data.J Am Coll Cardiol. 1997;30:1451–60.
