# Supplementary figures and images for: Assessment of Myocardial Viability in Ischemic Cardiomyopathy With Reduced Left Ventricular Function Undergoing Coronary Artery Bypass Grafting
Source: Clin Cardiol. 2024 Jul 2;47(7):e24307. doi: 10.1002/clc.24307 (PMC11217808; doi:10.1002/clc.24307)

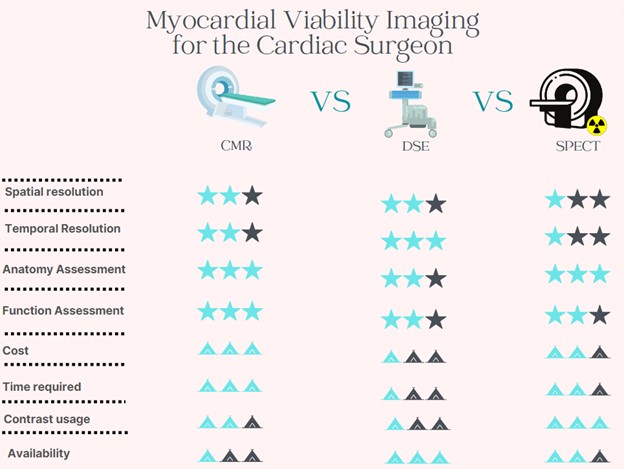

Supplement: Supplementary file 2 — Supporting information. [file CLC-47-e24307-s002.docx]
